# Author Correction: Capturing electron-driven chiral dynamics in UV-excited molecules

**DOI:** 10.1038/s41586-024-07676-7

**Published:** 2024-06-18

**Authors:** Vincent Wanie, Etienne Bloch, Erik P. Månsson, Lorenzo Colaizzi, Sergey Ryabchuk, Krishna Saraswathula, Andres F. Ordonez, David Ayuso, Olga Smirnova, Andrea Trabattoni, Valérie Blanchet, Nadia Ben Amor, Marie-Catherine Heitz, Yann Mairesse, Bernard Pons, Francesca Calegari

**Affiliations:** 1 Center for Free-Electron Laser Science CFEL, Deutsches Elektronen-Synchrotron DESY, Hamburg, Germany; 2 Université de Bordeaux - CNRS - CEA, CELIA, UMR5107, Talence, France; 3 Physics Department, Universität Hamburg, Hamburg, Germany; 4 The Hamburg Centre for Ultrafast Imaging, Universität Hamburg, Hamburg, Germany; 5 Department of Physics, Imperial College London, London, UK; 6 Max-Born-Institut, Berlin, Germany; 7 Technische Universität Berlin, Berlin, Germany; 8 Institute of Quantum Optics, Leibniz Universität Hannover, Hannover, Germany; 9 CNRS, UPS, LCPQ (Laboratoire de Chimie et Physique Quantiques), FeRMI, Toulouse, France; 10 Present Address: Department of Physics, Politecnico di Milano, Milano, Italy; 11 Present Address: School of Physical and Chemical Sciences, Queen Mary University of London, London, UK

**Keywords:** Excited states, Atomic and molecular interactions with photons, Ultrafast photonics, Attosecond science

Correction to: *Nature* 10.1038/s41586-024-07415-y Published online 22 May 2024

In the version of the article initially published, in the “Analysis of the VMIS images” section of the Methods, the sentence “The validity of the analysis protocol despite the lack of cylindrical symmetry induced by the anisotropy of excitation of the linearly polarized UV-pump pulse has been demonstrated in refs. 11,12,37” has been amended to read “A similar analysis protocol, ignoring the lack of cylindrical symmetry induced by the anisotropy of excitation of the linearly polarized UV-pump pulse, was used in refs. 11 and 12. It was recently shown that the harmonic terms describing symmetry breaking could be of significant amplitude^37^. In our experiment, they could be measured by repeating the measurements with a few different orientations of the pump polarization with respect to the detector plane, and tomographically reconstructing the 3D-PECD by Hankel transform as was done in ref. 37”. In the Extended Data Fig. 3 caption, a citation to ref. 37 has been removed. The changes are reflected in the HTML and PDF versions of the article.

