# Supplementary material for: Capturing electron-driven chiral dynamics in UV-excited molecules
Source: Nature. 2024 May 22;630(8015):109–15. doi: 10.1038/s41586-024-07415-y (PMC11153151; doi:10.1038/s41586-024-07415-y)
Supplement: Supplementary file 1 — Supplementary Sections 1–4, including Supplementary Figs. 1–12. [file 41586_2024_7415_MOESM1_ESM.pdf]

nature portfolio

https://doi.org/10.1038/s41586-024-07415-y

Supplementary information

Capturing electron-driven chiral dynamics in UV-excited molecules

In the format provided by the authors and unedited

Supplementary Information: *Capturing electron-driven chiral dynamics in UV-excited molecules*

Vincent Wanie*, Etienne Bloch, Erik P. Månsson, Lorenzo Colaizzi,
Sergey Ryabchuk, Krishna Saraswathula, Andres F. Ordonez, David Ayuso,
Olga Smirnova, Andrea Trabattoni, Valérie Blanchet, Nadia Ben Amor,
Marie-Catherine Heitz, Yann Mairesse, Bernard Pons*, Francesca Calegari*

We provide supplementary information on the numerical calculations which underlie our interpretation of oscillating photoelectron circular dichroism in terms of quantum beatings between transient Rydberg states and illustrate the concept of probe-induced forward/backward fragment asymmetry.

Contents

1 Electronic spectrum of methyl-lactate **5**

2 Pump-probe dynamics with fixed nuclei **12**

2.1 The excitation step 12

2.2 The ionization step 13

2.3 Convergence of the results and low energy photoelectron dynamics 18

3 Molecular dynamics calculations **20**

4 Probe-induced active orientation of the sample and forward/backward fragment asymmetry **26**

4.1 Probe-induced active orientation of the sample 26

4.2 Directed fragmentation and FBFA 28

List of Figures

S1 Equilibrium geometries of the neutral methyl-lactate molecule obtained using B3LYP (left) and LCBLYP (right) functionals to describe electron echange and correlation. These geometries are almost identical. The lenghts of the main bonds are given in Å. 5

S2 Energies of the excited states of (S)-methyl-lactate as a function of the state numbering index. The excited states stemming from HOMO (HOMO-1) excitation, with weight larger or equal to 0.5, are represented by red (blue) circles. The *nl* (or *n*) character of the Rydberg states is indicated, as well as the two vertical ionization potentials. The shaded black line is the two-photon spectral pump intensity, arbitrarily scaled in the x-direction. 7

S3 (a): Two-photon absorption (TPA) cross sections ($\circ$) of the excited states in the case of linearly polarized photons as a function of the binding energy E of the excited states. The dashed line is to guide the eye while the bold black one represents the two-photon spectral pump intensity $I_{2-UV}(E)$. (b): TPA cross sections convoluted with $I_{2-UV}(E)$. The blue, magenta and orange curves correspond to the spectral probe intensity, down-shifted in energy in order to elicit the transient Rydberg states leading to photoelectrons with energies $\epsilon = 0.05$, 0.25 and 0.5 eV through ionization by one photon centered at frequency $\omega = 1.75$ eV. The red and green filled circles indicate the excited states stemming from HOMO and HOMO-1 excitations, respectively, with weights $R_{HOMO,HOMO-1} \geq 0.8$. The red and green empty circles correspond to HOMO and HOMO-1 ex-

cited states, respectively, with weights $0.6 \leq R_{HOMO,HOMO-1} < 0.8$. The state located at $E = 9.036$ eV (blue empty circle) mainly stems from excitation of the HOMO-2 orbital of the ML ground state. Note that all the excited states lying higher than 9.6 eV will lead to photoelectron kinetic energies ϵ_{kin} not relevant for the experimental data. 9

S4 MP-PECD for photoelectron energy $\epsilon_{kin} = 0.5$ eV as a function of the pump-probe delay, resulting from calculations including HOMO excitation states with weights $R_{HOMO} \geq 0.6$ (red line) and 0.8 (blue line). 19

S5 (a): Experimental (blue line) and theoretical (green line) MP-PECD associated to low energy photoelectrons with $\epsilon = 0.05$ eV, as a function of the pump-probe delay. (b): Associated power spectra: calculations (green line), including HOMO excited states with weights larger or equal to 0.6, while the blue line corresponds to the experimental results. 19

S6 Equilibrium geometry of neutral (S)-methyl-lactate (top row). Three principal bonds (C-CH_3, C-C and O-H) are marked since they allow to distinguish the three isomers of the cation in its ground electronic state (bottom row) obtained at the CAM-B3LYP/6-311++G(dp) level. Internuclear distances are given in Å. 21

S7 Classical trajectories are launched on the potential energy surface of the ground state of the cation, with initial coordinates and momenta sorted from the Wigner distribution associated to the neutral molecule ground state geometry (a). 40% and 50% of these trajectories converge towards Isomer1 and Isomer2 of the cation within 60 fs, respectively (b). There is a strong electronic reorganization along Isomer2 trajectories, here pictured by the evolution of the hole wave-function, while the electronic configuration remains quite stable along Isomer1 trajectories (c). 22

S8 Temporal evolution of the excited state energies of neutral methyl-lactate along trajectories belonging to the Isomer1 (a) and Isomer2 (b) nuclear trajectory sets. The excited states are represented by black lines, while the evolution of the fundamental and first excited states of the cation are represented by the red and blue lines, respectively. 24

S9 Averaged orientation of ML cations upon ionization of a 3d-4p Rydberg wavepacket, $< \hat{\mathbf{e}}^{(h)}_{lab,\eta} >_{\hat{\mathbf{R}}} (\epsilon, t)$, for $\epsilon = 0.25$ eV (see eq. (18) and text) and $(h = +1, \eta = z)$: blue line, $(h = -1, \eta = z)$: dashed blue line, $(h = 0, \eta = z)$: black circles, $(h, \eta = x$ or $y)$: red squares, (h, η) for an achiral (H_2) system: green triangles. . 28

S10 Energy diagram for the ML cation and associated fragments from its corresponding Isomer2. The energies are obtained by CAM-B3LYP/6-311++G(dp) calculations and compared to energies (in brackets) resulting from CCSD(T) calculations. 29

S11 Illustration of a possible fragmentation process in the ML cation. The time reference ($t = 0$) is the ionization time of neutral ML. 30

S12 Dependence on ϵ of (a) $< \cos\theta >^{(+1)}_{\hat{\mathbf{R}}} (\epsilon, t) =< \hat{\mathbf{e}}_{lab,z} >^{(+1)}_{\hat{\mathbf{R}}} (\epsilon, t)$, measuring the preferential orientation along the light propagation axis of ML cations upon ionization and (b) $FBFA^{(+1)}(\epsilon, t)$, the forward/backward fragment asymmetry. 31

Atomic units are used throughout this section unless otherwise stated.

1 Electronic spectrum of methyl-lactate

The equilibrium geometry of (S)-methyl-lactate, shown in Fig. S1, has been obtained from Density Functional Theory (DFT, (*1*)) calculations using a so-called Dunning-Hay (double-zeta) underlying gaussian basis, including polarization and diffuse orbitals (*2, 3*), and hybrid B3LYP functional (*4, 5*) to describe electron exchange and correlation. The calculations have been performed using the GAMESS-US quantum chemistry package (*6*). Additionally to the equilibrium geometry, these calculations also provide a reliable description of the electronic ground state of the molecule whose vertical ionization potential I_P is 10.51 eV.

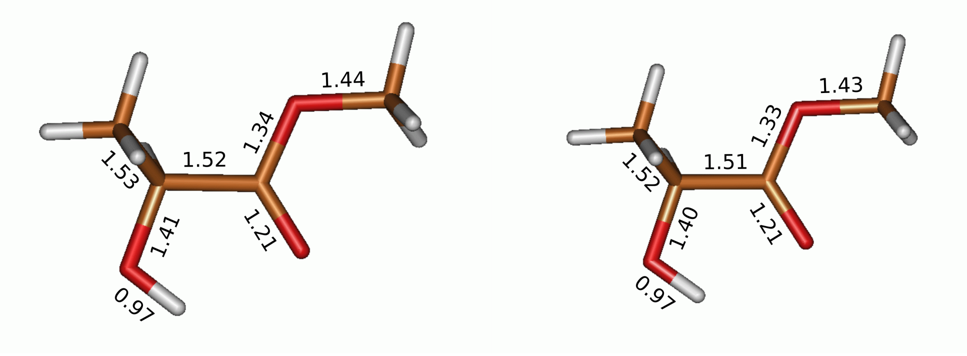

Fig. S1. Equilibrium geometries of the neutral methyl-lactate molecule obtained using B3LYP (left) and LCBLYP (right) functionals to describe electron echange and correlation. These geometries are almost identical. The lenghts of the main bonds are given in Å.

The experimental UV pump is spectrally centered about 4.7 eV with a typical full width at half maximum of 0.5 eV. The absorption of two pump photons thus promotes one electron onto high lying excited states, located about (9.4 ± 0.5) eV. Consistently with the former calculations, we aimed at describing these excited states by means of Time-Dependent DFT (TDDFT, (*7*)). However, the gaussian basis traditionally employed in quantum chem-

istry (see, e.g., (*8–10*)) are not designed for such high excitation energy with diffuse electronic wavefunctions. Therefore, we enlarged the Dunning-Hay basis by a set of contracted gaussian orbitals which fit the $n = 3 - 8$ states of atomic hydrogen. These orbitals have been obtained by diagonalizing the hydrogen Hamiltonian in a set of even-tempered primitive functions $g_{ilm}(\mathbf{r}) = \mathcal{N}_{il} r^l \exp\left(-\alpha_i r^2\right) Y_l^m(\hat{\mathbf{r}})$, where Y_l^m are spherical harmonics and $\mathcal{N}_{il}$ is a normalization factor. The α_i exponents lie within a geometrical series $\alpha_i = \alpha_0 \beta^p$ with $\alpha_0 = 2.5 \times 10^{-5}$, $\beta = 1.8$ and $0 \leq p \leq p_{max} = 22$ for all (l, m) angular symmetries. We have restricted the number of angular symmetries to $l \leq 4$ so that the diagonalization procedure amounts to contract the 23s23p23d23f23g primitive basis to 6s6p6d5f5g (since we retained only the states with $n = 3 - 8$, see Fig. S2). We first have checked the adequacy of our homemade enlarged basis by performing TDDFT calculations for the simple atomic system Li. We changed the B3LYP functional to the long-range corrected-BLYP (LCBLYP) one, which presents the expected Coulombic behaviour as the interelectronic distance tends to infinity (*11*). This feature is particularly important as one aims at describing electronic states which spread far away from the ionic core. These calculations have been found to reproduce accurately the high-lying Rydberg states of Li tabulated at NIST (*12*).

Subsequently, we performed TDDFT calculations for neutral methyl-lactate using our enlarged basis (centering the 6s6p6d5f5g functions on the center of charge of the cation), LCBLYP functional and the equilibrium geometry derived from our previous calculations. Note that there is no inconsistency in computing state properties using LCBLYP while the molecular geometry is optimized using B3LYP, since both functionals yield almost identical equilibrium geometries, as shown in Fig. S1. Even if DFT-based formalisms are based on electron density and not wavefunctions, the TDDFT excited states are usually interpreted in terms of linear combinations of single electron excitations from occupied ϕ_a to virtual ϕ_r orbitals on the basis of the

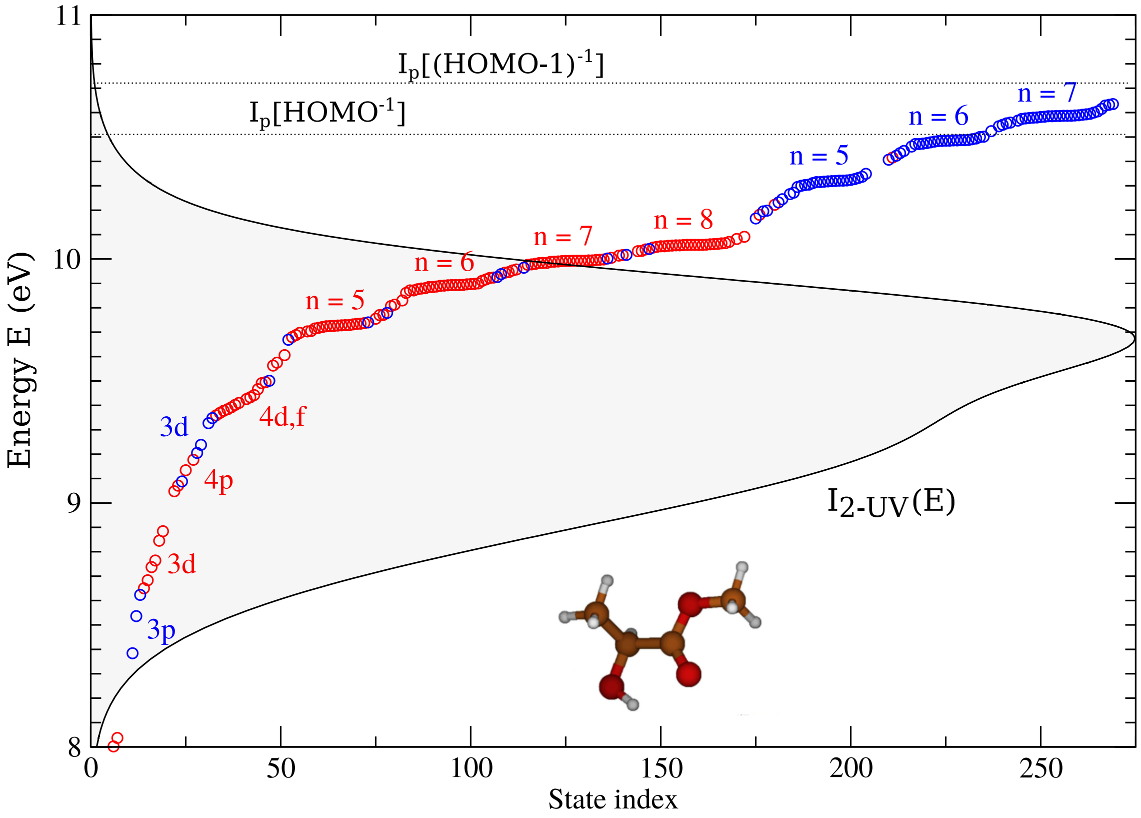

Fig. S2. Energies of the excited states of (S)-methyl-lactate as a function of the state numbering index. The excited states stemming from HOMO (HOMO-1) excitation, with weight larger or equal to 0.5, are represented by red (blue) circles. The *nl* (or *n*) character of the Rydberg states is indicated, as well as the two vertical ionization potentials. The shaded black line is the two-photon spectral pump intensity, arbitrarily scaled in the x-direction.

fundamental ground-state Slater determinant, according to:

$$\Psi_j = \sum_{a,r} c_{a\to r} \frac{1}{\sqrt{N!}} \begin{vmatrix} \phi_1(\mathbf{x}_1) & \dots & [\phi_a(\mathbf{x}_1) \to \phi_r(\mathbf{x}_1)] & \dots & \phi_n(\mathbf{x}_1) \\ \dots & \dots & \dots & \dots & \dots \\ \phi_1(\mathbf{x}_2) & \dots & [\phi_a(\mathbf{x}_2) \to \phi_r(\mathbf{x}_2)] & \dots & \phi_n(\mathbf{x}_2) \\ \dots & \dots & \dots & \dots & \dots \\ \phi_1(\mathbf{x}_N) & \dots & [\phi_a(\mathbf{x}_N) \to \phi_r(\mathbf{x}_N)] & \dots & \phi_n(\mathbf{x}_N) \end{vmatrix} \tag{1}$$

where $N = 56$ is the number of electrons and $\mathbf{x}_i$ are electronic coordinates including spin. De-excitation processes, inherent in the TDDFT approach, have been found to be negligible and are depreciated in (1). One can estimate the weight of excitation from ϕ_a in the formation of the electronic state Ψ_j by computing $R_a = \sum_r |c_{a\to r}|^2$. We found that most of the 235 excited states that we computed below the first ionization threshold present HOMO or HOMO-1 character, with $R_{HOMO,HOMO-1} \geq 0.5$, respectively. Furthermore, these states are mainly Rydberg states whose character is identified by means of the effective quantum number $n_{eff} = \sqrt{\frac{1}{2(I_P - E)}}$, where E is the energy of the excited state. Note that $n_{eff} \in \mathbb{R}$ is generally lower than n in a given n-shell because of the presence of the electronic ionic core (*13*). The main angular symmetry of the excited states is determined by limiting the expansion (1) to the main $a \to r$ excitation routes with fixed a (associated to $R_a \geq 0.5$) and by projecting the (renormalized) reduced expansion onto spherical harmonics – the origin of electron coordinates is fixed at the center of mass of the molecule in the projection procedure. We present in Fig. S2 the energies of the excited states as a function of the state numbering index. We discriminate between the states formed by HOMO and HOMO-1 excitations, and indicate their *nl* character. We superimpose to this graph the two-photon spectral pump intensity, which fits the (smoothed) experimental one, and observe that the pump excitation will principally lead to the population of Rydberg states stemming from HOMO excitation.

TDDFT provides oscillator strengths and two-photon absorption (TPA) tensors (*6, 14*). The oscillator strengths tailor one-photon absorption and are thus not relevant to the present study. However, it is worth mentionning that the one-photon absorption spectrum computed with our

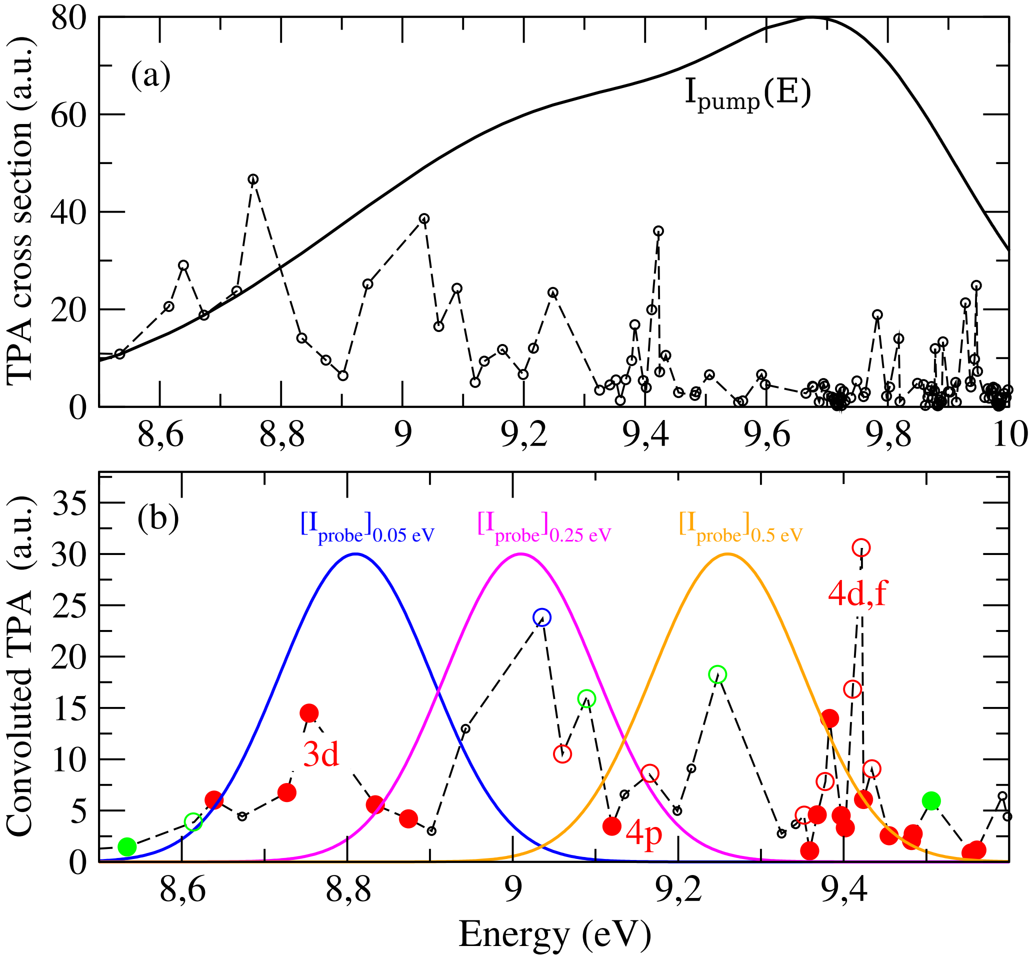

Fig. S3. (a): Two-photon absorption (TPA) cross sections (○) of the excited states in the case of linearly polarized photons as a function of the binding energy E of the excited states. The dashed line is to guide the eye while the bold black one represents the two-photon spectral pump intensity $I_{2-UV}(E)$. (b): TPA cross sections convoluted with $I_{2-UV}(E)$. The blue, magenta and orange curves correspond to the spectral probe intensity, down-shifted in energy in order to elicit the transient Rydberg states leading to photoelectrons with energies $\epsilon = 0.05$, 0.25 and 0.5 eV through ionization by one photon centered at frequency $\omega = 1.75$ eV. The red and green filled circles indicate the excited states stemming from HOMO and HOMO-1 excitations, respectively, with weights $R_{HOMO,HOMO-1} \geq 0.8$. The red and green empty circles correspond to HOMO and HOMO-1 excited states, respectively, with weights $0.6 \leq R_{HOMO,HOMO-1} < 0.8$. The state located at $E = 9.036$ eV (blue empty circle) mainly stems from excitation of the HOMO-2 orbital of the ML ground state. Note that all the excited states lying higher than 9.6 eV will lead to photoelectron kinetic energies ϵ_{kin} not relevant for the experimental data.

enlarged basis and LCBLYP functional nicely agrees with its experimental counterpart, which strengthens the accuracy of our TDDFT calculations – this will be shown in a forthcoming publication. More importantly, our description of perturbative pump excitation directly involves the TPA tensor elements. We thus present in Fig. S3(a) the TPA cross sections associated to a linearly polarized pump for the excited states lying in the 8.5-10 eV energy range. In Fig. S3(b), we emphasize the states which tailor the appearance of photoelectrons with energies $\epsilon = 0.05$, 0.25 and 0.5 eV in a framework where the vibrational energy is conserved during the one-photon probe-induced ionization – the reliability of this hypothesis will be verified in Section 3. In practice, this amounts to locate the spectral probe intensity about $I_P + \epsilon - \omega_P$ on the energy scale, $\omega_P = 1.75$ eV being the energy where the spectral probe intensity maximizes.

We focused in Fig. 3 of the main text on the excited states formed by (almost) exclusively HOMO excitation. In practice, these are the states fulfilling $R_{HOMO} \geq 0.8$, whose coherent superposition leads through ionization to the MP-PECD oscillations displayed in Fig. 3(b,d) of the main text. In Fig. S3(b), we proceed to a deeper analysis of all the excited states located in the 8.5-10 eV energy range of interest: we emphasize the states stemming from almost pure HOMO-1 excitation, fulfilling $R_{HOMO-1} \geq 0.8$, and also highlight the states with $0.6 \leq R_{HOMO,HOMO-1} \leq 0.8$. There is a state, located at $E = 9.036$ eV, whose main configuration corresponds to the excitation of the HOMO-2 orbital of the ML ground state. For all the states fulfilling $R_i \geq 0.8$, we approximate the multideterminal expression (1) by considering that excitation stems exclusively from orbital ϕ_i, yielding the simpler monoelectronic form $\Psi_j(\mathbf{r}) = \sum_b c_{i\to b}\phi_b(\mathbf{r})/R_i$, where the common electronic core has been factored out and omitted. This procedure is more questionable for $R_i < 0.6$ since excitation pathways with $j \neq i$ have a total weight comparable to the ith one. However, it has also been applied in that case to check the convergence of our results when the number of excited states included in the dynamical calculations increases (see Section 2.3).

One-photon ionization of two excited Rydberg states with distinct electronic cores leads to orthogonal continua. The ionization yield associated to their superposition thus consists of the incoherent sum of individual cross sections. This incoherent sum is stationnary in the frozen nuclei description of the interaction that we employ (and whose consistency will be checked in Section 3). In this framework, time-dependent PECD oscillations result from electronic coherences among transient excited Rydberg states with common electronic core. In this respect, the single HOMO-2 excited state is not amenable to PECD oscillations – it has not been considered in our calculations. Only two HOMO-1 excited states with $R_{HOMO-1} \geq 0.6$ and sizeable TPA cross sections show up in Fig. S3(b) – at $E = 9.090$ eV and 9.248 eV, respectively. However, one-photon ionization from these two states principally leads to photoelectrons with energies $\epsilon = 0.25$ eV and 0.5 eV, respectively. Therefore, these two excited states cannot explain the occurence of PECD oscillations at fixed ϵ's: they have not been considered in our dynamical calculations, as all the remaining HOMO-1 excited states of Fig. S3(b). Fortunately, very few states build on competing excitations of occupied orbitals, which would necessitate to take explicitely into account the multideterminantal nature of the excited states and associated electron continua. Furthermore, they generally have quite small TPA cross sections, except the one located at $E = 8.943$ eV. This latter state has however been neglected, as well as all the other (insignificant) states with complex electronic core. It is thus clear from Fig. S3(b) that most of the excited states of interest stem from excitation of the HOMO, and belong to the 3d, 4p and 4d,f Rydberg subshells. Photoelectrons with $\epsilon \sim 0.05$ eV arise from the coherent superposition of intermediate 3d Rydberg states while higher energy ($\epsilon \sim 0.5$ eV) photoelectrons result from the coherent mixing of 4p and 4d-f states. In between ($\epsilon \sim 0.25$ eV), photoelectrons emerge as the result of ionization from the 3d-4p coherent state superposition induced by the pump. We have discussed in the main text how these coherent superpositions of intermediate bound states lead to the appearance of PECD oscillations with ~ 15 fs period. This period corresponds to

the ~ 0.3 eV energy separation of the Rydberg subshells illustrated in Fig. S3(b).

2 Pump-probe dynamics with fixed nuclei

We consider adjacent and non-overlaping pump and probe pulses so that ionization occurs once the pump pulse has vanished. This allows a simpler description of the whole dynamics in terms of sequential excitation and ionization processes.

2.1 The excitation step

The intensity of the pump pulse is low so that we can describe the two-photon absorption by means of a second-order perturbative approach. We assume that the pump pulse is linearly polarized along $\hat{\mathbf{x}}$. The transition amplitude to the excited state i is directly proportional to the two-photon tensor element $T_{xx}^{(i)}$. It is convenient to define the polarization unit vector in terms of spherical coordinates such that $T_{xx}^{(i)} = \sum_{\rho_1,\rho_2} a_{\rho_1,\rho_2} T_{\rho_1,\rho_2}^{(i)}$ with $\rho_{1,2} \in \{-1, 0, 1\}$, $a_{1,1} = 1/2$, $a_{-1,-1} = 1/2$ and $a_{-1,1} = -1$ (all other $a_{i,j} = 0$). We consider a sample of randomly oriented molecules wherein each molecular orientation $\hat{\mathbf{R}}$ is defined in terms of Euler angles (α, β, γ). We thus passively rotate the polarization into the molecular frame by means of the Wigner rotation matrix $D_{q_i,\rho_i}^{(1)}(\hat{\mathbf{R}})$ according to $\hat{\rho}_i = \sum_{q_i} D_{q_i,\rho_i}^{(1)}(\hat{\mathbf{R}})\hat{q}_i$ (*15*). The transition amplitude for a fixed $\hat{\mathbf{R}}$ thus reads $T_{xx}^{(i)} = \sum_{\rho_1,\rho_2} \sum_{q_1,q_2} a_{\rho_1,\rho_2} D_{q_1,\rho_1}^{(1)}(\hat{\mathbf{R}}) D_{q_2,\rho_2}^{(1)}(\hat{\mathbf{R}}) T_{q_1 q_2}^{(i),mol}$, where $T_{q_1 q_2}^{(i),mol}$ is defined in the molecular frame and deduced from the previous TDDFT calculations. For sake of conciseness, we shall omit the mol superscript in what follows.

Many excited states can be reached through the pump excitation (see Figs. S2 and S3). This leads to a bound electron wavepacket whose expression in the molecular frame is, at time t after the pump vanishes:

$$\Phi(\hat{\mathbf{R}}, \mathbf{r}, t) = \sum_i \mathcal{A}_i(\hat{\mathbf{R}}) \Psi_i(\mathbf{r}) \exp(-iE_i t) \quad (2)$$

where E_i is the energy of the excited state Ψ_i and $\mathcal{A}_i(\hat{\mathbf{R}})$ consist of the two-photon transition amplitudes weighted by the square root of the two-photon spectral intensity of the pump, i.e. $\mathcal{A}_i(\hat{\mathbf{R}}) = \sqrt{I_{2-UV}(E_i)} \sum_{\rho_1,\rho_2} \sum_{q_1,q_2} a_{\rho_1,\rho_2} D^{(1)}_{q_1,\rho_1}(\hat{\mathbf{R}}) D^{(1)}_{q_2,\rho_2}(\hat{\mathbf{R}}) T^{(i)}_{q_1 q_2}$. The underlying two-photon absorption tensor elements $T^{(i)}_{xx}$ are real so that $\mathcal{A}_i(\hat{\mathbf{R}}) \in \mathbb{R}$ for all $\hat{\mathbf{R}}$'s and i's, up to a common phase factor which is not important.

Remind that we include in our treatment, according to our previous discussion, only the states stemming from mainly pure HOMO excitation (with $R_{HOMO} \geq 0.8$ in the main text and $R_{HOMO} \geq 0.6$ below).

2.2 The ionization step

In our modeling, the probe starts to act at the end of the pump, corresponding to $t = 10$ fs when estimating the temporal profiles of the pump and probe pulses by inverse Fourier transforms of the spectral shapes of Extended Data Fig. 1.

In the molecular frame, the ionization dipole associated to the ejection of a photoelectron with momentum $\mathbf{k}'$ (associated to the photoelectron energy $\epsilon_{kin} = k'^2/2$) reads

$$d^{mol}_{\mathbf{k}'}(\hat{\mathbf{R}}, t) = \sum_i \mathcal{A}_i(\hat{\mathbf{R}}) \sqrt{I_{1-NIR}(\omega_i)} < \Psi^{(-)}_{\mathbf{k}'} | \hat{\mathbf{e_h}}.\mathbf{r} | \Psi_i > \exp(-iE_i t) \tag{3}$$

where $I_{1-NIR}(\omega_i)$ is the spectral intensity profile of the probe pulse, with $\omega_i = k'^2/2 + I_p - E_i$, $\hat{\mathbf{e_h}}$ is the circular polarization state of the probe in the laboratory frame ($h = \pm 1$) and $\Psi^{(-)}_{\mathbf{k}'}(\mathbf{r})$ is the ingoing scattering state associated to the electron ejected into the continuum. Neither the scattering state nor the excited states explicitely depend on t in (3) since all the calculations are made assuming that the molecular nuclei remain frozen at their equilibrium locations at all t.

The scattering state is expanded onto partial waves

$$\Psi^{(-)}_{\mathbf{k}'}(\mathbf{r}) = \sum_{l,m} i^l e^{-i\sigma_{k'l}} \Psi^{(-)}_{k'lm}(\mathbf{r}) Y^{m*}_l(\hat{\mathbf{k}}') \tag{4}$$

where $\sigma_{k'l}$ are the asymptotic Coulomb phase shifts and $\Psi^{(-)}_{k'lm}$ are complex ingoing states with fixed (l, m) angular symmetries (*16*). Defining the probe polarization in the molecular frame where the wavefunctions are defined, and performing the inverse rotation to pass $Y_l^{m*}(\hat{\mathbf{k}'})$ from the molecular frame to the laboratory one, we obtain the ionization dipole in the laboratory frame for a fixed orientation $\hat{\mathbf{R}}$:

$$d^{(h)}_{\mathbf{k}}(\hat{\mathbf{R}}, t) = \sum_{i,l,m,\nu,\mu} \mathcal{A}_i(\hat{\mathbf{R}})\sqrt{I_{1-NIR}(\omega_i)}(-i)^l e^{i\sigma_{kl}} D^{(1)}_{\nu,h}(\hat{\mathbf{R}}) a_{iklm\nu} D^{(l)^*}_{m,\mu}(\hat{\mathbf{R}}) Y_l^{\mu}(\hat{\mathbf{k}}) \exp(-iE_i t) \tag{5}$$

where $a_{iklm\nu} =< \Psi^{(-)}_{klm}|\hat{\mathbf{e}_{\nu}}.\mathbf{r}|\Psi_i >$ are the state-selective continuum partial wave amplitudes. Note that the modulus of the wavevector is invariant under rotation, i.e. $k = k'$.

To evaluate the dipolar amplitudes $a_{iklm\nu}$, we expand $\Psi^{(-)}_{klm}$ onto real states according to:

$$\Psi^{(-)}_{klm}(\mathbf{r}) = \sum_{l',m'} (\mathbf{I} + i\mathbf{K})_{lm,l'm'} \Psi_{kl'm'}(\mathbf{r}) \tag{6}$$

where $\mathbf{I}$ is the unitary matrix and $\mathbf{K}$ is the so-called $\mathbf{K}$-matrix (*16*). The states $\Psi_{kl'm'}(\mathbf{r})$ are solutions of the Schrödinger equation $\left(-\frac{1}{2}\nabla^2 + V(\mathbf{r}) - \epsilon\right)\Psi_{kl'm'}(\mathbf{r}) = 0$. The potential V is split as $V(\mathbf{r}) = V_{e-n}(\mathbf{r}) + V^{(d)}_{e-e}(\mathbf{r}) + V^{(ex)}_{e-e}(\mathbf{r})$, where $V_{e-n}(\mathbf{r})$ is the electron-nucleus potential while $V^{(d)}_{e-e}(\mathbf{r})$ and $V^{(ex)}_{e-e}(\mathbf{r})$ are the direct (Hartree) and exchange parts of the electron-electron interaction, respectively. We define the direct interaction part as $V^{(d)}_{e-e}(\mathbf{r}) = \int \frac{\rho(\mathbf{r}')}{|\mathbf{r}-\mathbf{r}'|} d\mathbf{r}'$ and employ for exchange the Xα statistical form $V^{(ex)}_{e-e}(\mathbf{r}) = -\frac{3}{2}\alpha\left(\frac{3\rho(\mathbf{r})}{\pi}\right)^{1/3}$ with $\alpha = 0.75$ (*17*). $\rho(\mathbf{r})$ corresponds to the total electronic density of the molecule in its ground state. It may seem counterintuitive to employ this density to describe ionization processes occurring from Rydberg states. However the Xα approach has been designed so that all electrons move in an average potential field, no matter of the electron (and orbital location) whose motion is investigated (*18*). This has been established for electrons belonging to the ground state and we extrapolate herein the feature to excited systems, considering that one-electron Rydberg excitation does not significantly affect the whole ionic potential. However, the po-

tential $V(\mathbf{r})$ does not present the expected $-1/r$ asymptotic behaviour. We remedy this by implementing the Latter correction (*19*): $V(r \geq r_0) = -1/r$, with r_0 such that $V(r_0) = -1/r_0$, in all $\hat{\mathbf{r}}$ directions. Subsequently to a single-center decomposition of $V(\mathbf{r})$ onto (real) spherical harmonics, the Schrödinger equation is solved using the remormalized Numerov method (*20*). The $\mathbf{K}$-matrix elements are found by matching the computed $\Psi_{klm}(\mathbf{r})$ states to $\frac{1}{\sqrt{\pi k r}} \sum_{l',m'} \left(\sin(\theta_{l'})\delta_{ll'}\delta_{mm'} + \cos(\theta_{l'})K_{lm,l'm'}\right) Y_{l'}^{m'}(\hat{\mathbf{r}})$ in the $r \to \infty$ asymptotic region, with $\theta_{l'} = kr - l'\pi/2 - (1/k)\ln(2kr) + \sigma_{kl'}$. This allows to completely define the ingoing scattering states (6) as well as the state-selective continuum partial wave amplitudes $a_{iklm\nu}$ which define the ionization dipole (5).

For a single molecular orientation, the differential ionization cross section is directly proportional to $|d_{\mathbf{k}}^{(h)}(\hat{\mathbf{R}}, t)|^2$ according to the Fermi golden rule. However, the molecules are randomly oriented within the sample so that the total signal is, for a given probe pulse helicity h:

$$\frac{d\bar{\sigma}^{(h)}}{d\Omega_{\mathbf{k}}}(k, \theta, \varphi, t) \propto \frac{1}{8\pi^2} \int d\hat{\mathbf{R}} |d_{\mathbf{k}}^{(h)}(\hat{\mathbf{R}}, t)|^2. \tag{7}$$

where (θ, φ) are the spherical angles characterizing the direction of electron ejection. $\theta = \cos^{-1}(\mathbf{k}.\hat{\mathbf{z}})$ where $\hat{\mathbf{z}}$ is the unit vector pointing towards the pulse propagation axis. Using eqs. (2)-(7) and following the algebra thorougly detailed in (*21*), the orientation-averaged cross section can be put in the closed form:

$$\frac{d\bar{\sigma}^{(h)}}{d\Omega_{\mathbf{k}}}(k, \theta, \varphi, t) = \sum_{s=0}^{6} \sum_{i=-2}^{2} b_{s,2i}^{(h)}(k, t) Y_s^{2i}(\theta, \varphi) \tag{8}$$

where

$$
\begin{aligned}
b_{s,2i}^{(h)}(k,t) &= \frac{1}{2s+1}\sum_{\substack{\rho_1,\rho_2\\ \rho_1',\rho_2'}} a_{\rho_1,\rho_2}a_{\rho_1',\rho_2'}\sum_{\substack{q_1,q_2\\ q_1',q_2'}}(-1)^{q_1'+q_2'}\sum_{Q_1,Q_2=0}^{2} < 1\; q_1, 1\; -q_1'|Q_1\; q_1-q_1' > \times \\
&< 1\;\rho_1, 1\; -\rho_1'|Q_1\;\rho_1-\rho_1' >< 1\; q_2, 1\; -q_2'|Q_2\; q_2-q_2' > \times \\
&< 1\;\rho_2, 1\; -\rho_2'|Q_2\;\rho_2-\rho_2' > \sum_{K=|Q_1-Q_2|}^{Q_1+Q_2} < Q_1\; q_1-q_1', Q_2\; q_2-q_2'|K\; q_1+q_2-q_1'-q_2' > \times \\
&< Q_1\;\rho_1-\rho_1', Q_2\;\rho_2-\rho_2'|K\; -2i > \sum_{\nu,\nu'=-1}^{1}\sum_{j=0}^{2}(-1)^j < 1\;\nu, 1\; -\nu'|j\;\nu-\nu' > \times \qquad (9) \\
&< 1\; -h, 1\; h|j\; 0 >< K\; q_1+q_2-q_1'-q_2', j\;\nu-\nu'|s\; q_1+q_2-q_1'-q_2'+\nu-\nu' > \times \\
&< K\; -2i, j\; 0|s\; -2i > \sum_{\substack{l,m\\ l',m'}}\sqrt{\frac{(2l+1)(2l'+1)}{4\pi(2s+1)}}(-1)^{\nu+m'} < l\; 0, l'\; 0|s\; 0 > \times \\
&< l\; -m, l'\; m'|s\; m'-m > \delta_{q_1+q_2-q_1'-q_2'+\nu-\nu',m-m'}(-i)^{l-l'}e^{i(\sigma_{kl}-\sigma_{kl'})}\sum_{n,n'} a_{nklm\nu} \times \\
&\quad a^*_{n'kl'm'\nu'}T^{(n)}_{q_1,q_2}T^{(n')*}_{q_1',q_2'}\sqrt{I_{2-UV}(E_n)I_{2-UV}(E_{n'})I_{1-NIR}(\omega_n)I_{1-NIR}(\omega_{n'})}e^{-i(E_n-E_{n'})t}.
\end{aligned}
$$

The cross section is obviously real so that $b_{l,2i}^{(h)} = b_{l,-2i}^{(h)*}$ (the $b_{l,0}^{(h)}$ coefficients are thus real). Therefore, the orientation-averaged cross section can be written as

$$
\frac{d\bar{\sigma}^{(h)}}{d\Omega_{\mathbf{k}}}(k,\theta,\varphi,t) = \sum_{s=0}^{6}\sum_{i=-2}^{2}\tilde{b}_{s,2i}^{(h)}(k,t)\tilde{Y}_s^{2i}(\theta,\varphi) \qquad (10)
$$

where $\tilde{Y}_s^{2i}$ are real spherical harmonics. Those with $2i < 0$ are associated to sine real harmonics while those with $2i \geq 0$ correspond to cosine ones (*22*). The $\tilde{b}_{s,2i}^{(h)}(k,t)$ coefficients are related to the $b_{s,2i}^{(h)}(k,t)$ ones through $\tilde{b}_{s,2i}^{(h)}(k,t) = \sqrt{2}\mathcal{R}[b_{s,2i}^{(h)}(k,t)]$ if $i > 0$ and $\tilde{b}_{s,2i}^{(h)}(k,t) = -\sqrt{2}\mathcal{I}[b_{s,2i}^{(h)}(k,t)]$ if $i < 0$. The enantio-selective coefficients are $\tilde{b}_{2p+1,2i}^{(h)}$, with $0 \leq p \leq 2$ and $0 \leq i \leq 2$, and $\tilde{b}_{2p,-2i}^{(h)}$ with $1 \leq p \leq 3$ and $1 \leq i \leq 2$.

The antisymmetric chirosensitive part $\mathcal{A}(k,\theta,\varphi,t)$ of the photoelectron angular distribution is obtained by substracting the differential cross sections associated to $h = +1$ and $h = -1$,

corresponding to left and right circularly polarized ionizing radiations,

$$\begin{aligned}\mathcal{A}(\mathbf{k},t) &= \frac{d\bar{\sigma}^{(+1)}}{d\Omega_{\mathbf{k}}}(k,\theta,\varphi,t) - \frac{d\bar{\sigma}^{(-1)}}{d\Omega_{\mathbf{k}}}(k,\theta,\varphi,t) \\ &= \sum_{s=0}^{2}\sum_{i=-2}^{2}\mathcal{R}[b^{(+1)}_{2s+1,2i}(k,t)]Y^{2i}_{2s+1}(\theta,\varphi) + \sum_{s=1}^{2}\sum_{\substack{i=-2\\ i\neq 0}}^{2}\mathcal{I}[b^{(+1)}_{2s,2i}(k,t)]Y^{2i}_{2s}(\theta,\varphi).\end{aligned} \tag{11}$$

However, it is convenient to define an angularly-integrated asymmetry, to which one usually refer to as multiphotonic PECD (MP-PECD, (*23*)), as twice the difference of electrons emitted in the forward and backward hemispheres for $h = +1$, normalized to the average number of electrons collected in one hemisphere:

$$\begin{aligned}MP-PECD(k,t) &= 2\frac{\int_0^{2\pi}\int_0^{\pi/2}\sin(\theta)\left(\frac{d\bar{\sigma}^{(+1)}}{d\Omega_{\mathbf{k}}}(k,\theta,\varphi,t) - \frac{d\bar{\sigma}^{(+1)}}{d\Omega_{\mathbf{k}}}(k,\pi-\theta,\varphi,t)\right)d\theta d\varphi}{\frac{1}{2}\int_0^{2\pi}\int_0^{\pi/2}\sin(\theta)\left(\frac{d\bar{\sigma}^{(+1)}}{d\Omega_{\mathbf{k}}}(k,\theta,\varphi,t) + \frac{d\bar{\sigma}^{(+1)}}{d\Omega_{\mathbf{k}}}(k,\pi-\theta,\varphi,t)\right)d\theta d\varphi} \\ &= \frac{1}{b^{(+1)}_{0,0}(t)}\left(2\sqrt{3}b^{(+1)}_{1,0}(k,t) - \frac{\sqrt{7}}{2}b^{(+1)}_{3,0}(k,t) + \frac{\sqrt{11}}{4}b^{(+1)}_{5,0}(k,t)\right).\end{aligned} \tag{12}$$

Eq. (12) reduces to the well-known expression $\frac{1}{b^{(+1)}_{0,0}(t)}\left(2b^{(+1)}_{1,0}(k,t) - \frac{1}{2}b^{(+1)}_{3,0}(k,t) + \frac{1}{4}b^{(+1)}_{5,0}(k,t)\right)$ when the laser-molecule interaction fulfills cylindrical symmetry and $\frac{d\bar{\sigma}^{(h)}}{d\Omega_{\mathbf{k}}}(k,\theta,\varphi,t)$ is decomposed onto Legendre polynomials $P_l(\cos(\theta))$ since $Y^0_l(\theta,\varphi) = \sqrt{\frac{2l+1}{4\pi}}P_l(\cos(\theta))$ (*24*). Note that the latter expression has been used in the interpretation of the present raw experimental data, as discussed in (*24*).

Note that the PECD arises as the result of the interferences of continuum partial waves (l, m) and (l', m') with $l \neq l'$ and $m \neq m'$ (*25*) encoded in the $a_{nklm\nu}a_{n'kl'm'\nu'}$ term in equation (9). These interferences do not show up in the total cross section, associated to $b_0(\epsilon, t)$, since if $s = 0$ in (9), $l = l'$ because of the $< l\ 0, l'\ 0|s\ 0 >$ Clebsch-Gordan coefficient and $m = m'$ for $< l\ -m, l'\ m'|s\ m' - m >$ to be non zero.

2.3 Convergence of the results and low energy photoelectron dynamics

The computed MP-PECD are reported in Fig. 3 of the main text for wavevector amplitudes associated to photoelectron energies $\epsilon_{kin} = 0.25$ and 0.5 eV. These results have been obtained by means of calculations which include the excited states built on almost pure HOMO excitation, characterized by $R_{HOMO} \geq 0.8$ (see Fig. S3(b)). The MP-PECD oscillations have been attributed to quantum beatings between these states. Therefore, it is worth investigating to what extent these oscillations survive when the number of excited states is enlarged, by decreasing the R_{HOMO} minimal value. We observe in Fig. S3(b) that when decreasing R_{HOMO} down to 0.6, all the three 4p states show up and the 4d-f subshells are also further filled. The states excited from HOMO-1 are still insignificant, as discussed in Section 1. Dynamical calculations have been performed for $\epsilon = 0.5$ eV, associated to a bound spectral range where the number of probed excited states is particularly increased when $R_{HOMO} \geq 0.6$ (see Figs. S3(b)). The resulting MP-PECD is presented in Fig. S4 and compared to the one obtained when $R_{HOMO} \geq 0.8$. Even if the magnitude of the MP-PECD is sensitively affected by the minimal R_{HOMO} value, the oscillations remain with a main frequency which is characteristic of quantum beating between the 4p and 4d-f Rydberg subshells. Additional intra-shell beatings occur, leading to the change observed at short delays in Fig. S4. However, this figure shows that our description of coherent MP-PECD is robust and safely survives as the electronic spectrum considered is enlarged.

We present in Fig. S5(a) the comparison of experimental and theoretical MP-PECD for the lowest photoelectron energy band (see Fig. 2 of the main text). ϵ has been set to 0.05 eV in the calculations which employ the last $R_{HOMO} \geq 0.6$ criterion. The agreement is not as good as for the highest energy bands (see Fig. 3 of the main text): while the experiment provides MP-PECD oscillations with ~ 15 fs period, associated to ~ 0.3 eV frequency in Fig. S5(b), the calculations yield mainly intra-shell 3d beating at low frequencies, accompanied by a small

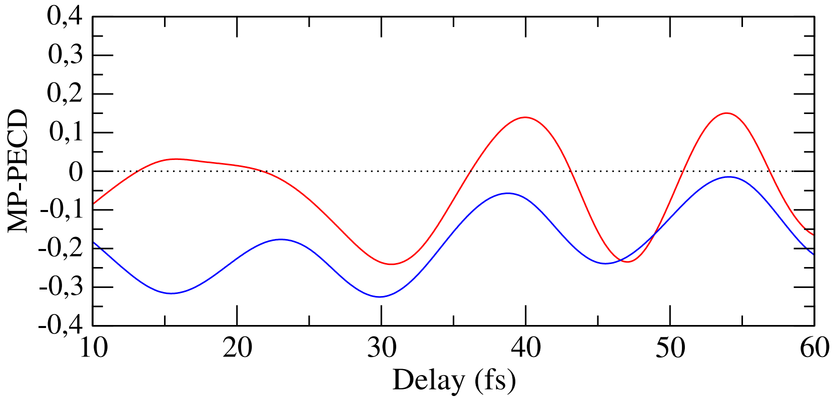

Fig. S4. MP-PECD for photoelectron energy $\epsilon_{kin} = 0.5$ eV as a function of the pump-probe delay, resulting from calculations including HOMO excitation states with weights $R_{HOMO} \geq 0.6$ (red line) and 0.8 (blue line).

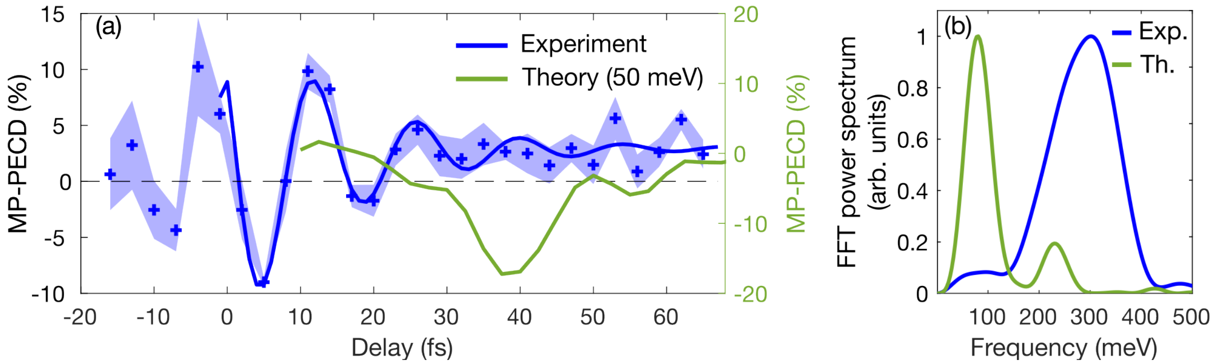

Fig. S5. (a): Experimental (blue line) and theoretical (green line) MP-PECD associated to low energy photoelectrons with $\epsilon = 0.05$ eV, as a function of the pump-probe delay. (b): Associated power spectra: calculations (green line), including HOMO excited states with weights larger or equal to 0.6, while the blue line corresponds to the experimental results.

contribution of 3d-4p beating around 0.25 eV. The reason for this discrepancy may be twofold: (i) the Xα description of the electronic continuum may be inacurrate so close to the ionization threshold – electron-electron correlation beyond the Xα framework are generally important in this range, (ii) the multi-determinantal state located around 9 eV, not included in our description (see Fig. S3(b)), may play a role in the dynamics. However, these last results still illustrate how coherent electron motion in excited states leads to fast modulations of the MP-PECD.

Up to now, all the results, including those presented in the main text, have been obtained with methyl-lactate molecules frozen at their equilibrium geometry. We check the consistency of this assumption in the next section.

3 Molecular dynamics calculations

Describing full vibronic dynamics among tens of electronic states in a high energy range is beyond state-of-the-art calculations. Therefore, we assume that the Rydberg states share the same nuclear dynamics than the cation to which they correlate. This means that monitoring nuclear dynamics along the cation states gives a global glance at the nuclear dynamics taking place in the Rydberg states. Here we focus on the cation in its electronic ground state since the Rydberg states populated by the pump pulse stem mainly from excitation of the HOMO of the neutral molecule.

Geometry optimizations at the CAM-B3LYP/6-311++G(dp) (*26–28*) level yield three minima of the cation, which are illustrated in Fig. S6 and compared to the one of the neutral molecule. Note that using CAM-B3LYP instead of LCBLYP is not an issue since both functionals yield almost identical geometries, similarly to the case of neutral molecules illustrated in Fig. S1. The first cationic isomer, to which we refer to as Isomer1 in Fig. S6, is very similar to the neutral molecule in spite of a slightly elongated C-CH_3 bond (from 1.523 to 1.683 Å). The second isomer (Isomer2) lies 0.41 eV below the first one and basically corresponds to a

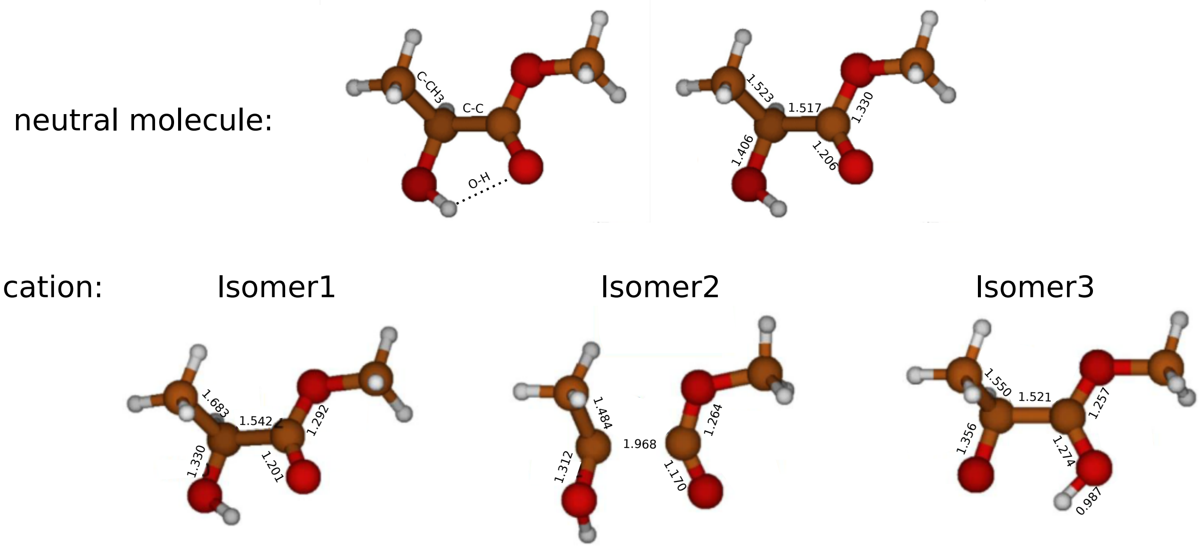

Fig. S6. Equilibrium geometry of neutral (S)-methyl-lactate (top row). Three principal bonds ($C\text{-}CH_3$, C-C and O-H) are marked since they allow to distinguish the three isomers of the cation in its ground electronic state (bottom row) obtained at the CAM-B3LYP/6-311++G(dp) level. Internuclear distances are given in Å.

breaking of the central C-C bond. Finally, the third isomer (Isomer3) is associated to hydrogen transfer from the oxygen atom to the carbonyl group and lies 0.54 eV below the first minimum.

To see how these three isomers are reached in the dynamics, we performed classical molecular dynamics calculations, in terms of 250 nuclear trajectories evolving on the fundamental potential energy surface of the cation (at the the CAM-B3LYP/6-311++G(dp) level) and propagated using the Newton-X package (*29, 30*) interfaced with Gaussian09 (*31*). The adiabatic trajectories have been integrated with a 0.5 fs time step until maximum 100 fs. Initial coordinates and momenta of the trajectories have been taken from a Wigner distribution of the (harmonic) vibrational ground state of the neutral molecule in its electronic ground state. Early time dynamics, restricted to $t \leq 60$ fs, can be analyzed by considering the time evolution of the three interatomic distances charactering the three cationic isomers, i.e. the lateral $C\text{-}CH_3$, central C-C and transfer O-H coordinates. Among the 250 trajectories, only 10% converge di-

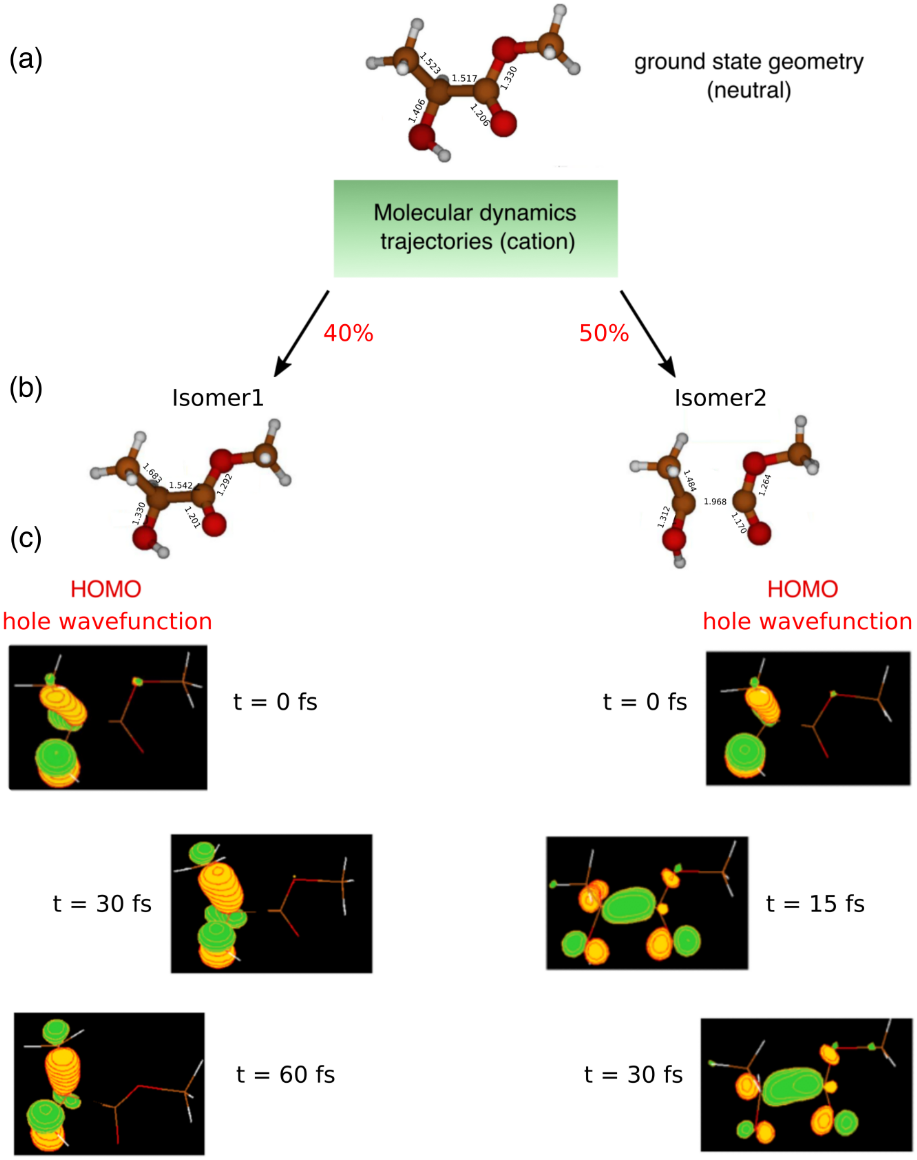

Fig. S7. Classical trajectories are launched on the potential energy surface of the ground state of the cation, with initial coordinates and momenta sorted from the Wigner distribution associated to the neutral molecule ground state geometry (a). 40% and 50% of these trajectories converge towards Isomer1 and Isomer2 of the cation within 60 fs, respectively (b). There is a strong electronic reorganization along Isomer2 trajectories, here pictured by the evolution of the hole wavefunction, while the electronic configuration remains quite stable along Isomer1 trajectories (c).

rectly to Isomer3 with proton transfer occuring most of the time after 40 fs. The contribution of these few trajectories can be depreciated. About 50% of the trajectories exhibit a central C-C bond extending beyond 2 Å within 20-60 fs; they correlate to Isomer2. The rest (40%) of the trajectories shows slightly increasing C-CH3 bond as for Isomer1. The two main subsets of trajectories are illustrated in Fig. S7(b).

For the trajectories correlating directly to Isomer2, the electronic structure undergoes a strong reorganization. This is pictured in Fig. S7(c) by observing the shape of the HOMO of the neutral molecule along representative nuclear trajectories. Indeed, this orbital corresponds to the electron hole in the Rydberg states formed by the pump pulse, and promoted to the cation through ionization. We observe in Fig. S7(c) that the hole translates to the central C-C bond within the first tens of femtoseconds, related to the elongation of the central C-C bond inherent to these trajectories. On the contrary, the electronic configuration along representative trajectories correlating to Isomer1 remains quite stable during 60 fs – the hole density is approximately stationnary within this time interval in the illustration of Fig. S7(c). Therefore, the nuclear geometry changes only a little bit along Isomer1 trajectories so that a frozen nuclei description of the electron dynamics seems well suited for these trajectories.

One must refrain to conclude from Fig. S7 that the frozen nuclei description is wrong for $\sim 50\%$ of the dynamics, at least with respect to electronic coherences. We report in Fig. S8 the temporal evolution of the Rydberg state energies of neutral methyl-lactate along trajectories belonging to the Isomer1 and Isomer2 sets. We observe in both graphs that the Rydberg states associated to HOMO and HOMO-1 excitations remain roughly, and respectively, parallel to each other. Even if a closer look at the graphs indicates that the states pseudocross or even intersect, this means that electronic coherences, whose periods are dictated by $2\pi/\Delta E$, can survive the nuclear evolution with ΔE fixed at its $t = 0$ value. Moreover, the graphs also include the energies of the fundamental and first excited states of the cation. The two Rydberg

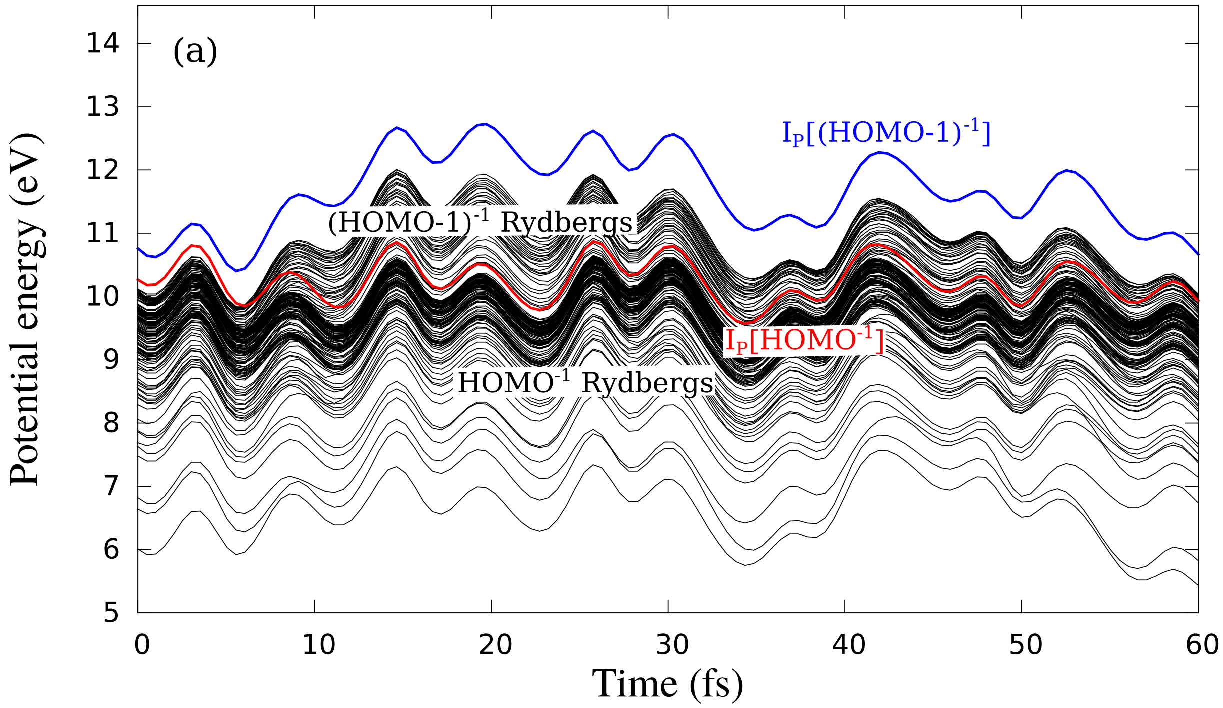

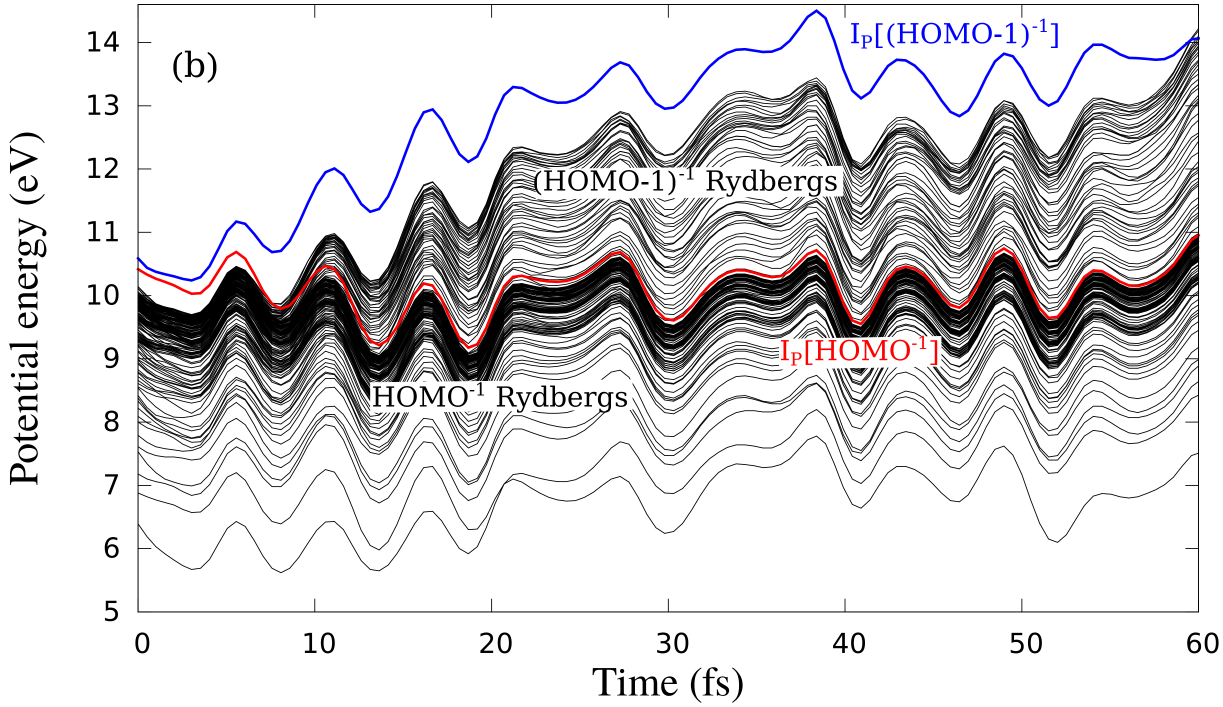

Fig. S8. Temporal evolution of the excited state energies of neutral methyl-lactate along trajectories belonging to the Isomer1 (a) and Isomer2 (b) nuclear trajectory sets. The excited states are represented by black lines, while the evolution of the fundamental and first excited states of the cation are represented by the red and blue lines, respectively.

groups are found to evolve similarly to the cationic state to which they correlate: this supports the propensity rule that states that the vibrational energy is conserved during the ionization step – this rule has been used in the identification of the Rydberg states leading to photoelectrons with fixed energy ϵ_{kin} (see Fig. S3(b)). Therefore, we have two groups of nuclear trajectories within which electronic coherences can be maintained for rather long time and easily probed by ionization. Coherence between the groups is impeded by the different nuclear conformations – from a quantum mechanical point of view, the two groups represent weakly overlapping vibrational wavepackets (see (*32*) and references therein). This leads us to ask why the measurements yield decreasing amplitude MP-PECD oscillations, which sign a loss of electronic coherence, while the frozen-nuclei description, globally validated by the molecular dynamics calculations, lead to coherences quantitatively maintained at all times. The decrease of the overlap of the nuclear wavepackets on the Rydberg states, as well as their dephasing, can not be totally ruled out, especially for large-scale systems such as ML which has 29 vibrational degrees of freedom (*33*). However, we venture that the electronic coherence mainly decreases because of non-adiabatic dynamics, not only between the states populated by the pump but also with lower-lying Rydberg and valence states. This last relaxation mechanism, referred to as internal conversion, appears in the experimental total photoelectron yield $b_0(\epsilon, t)$ which starts to decrease from delays as short as a few fs (see Extended Data Fig. 5). Even if non-adiabatic transitions have been found to preserve or even transfer electronic coherence between electronic states (*34, 35*), it is here highly probable that transfer from Rydberg to valence states, followed by the decrease of the phase-space overlap of nuclear wavepackets involving many vibrational modes (*33*), progressively makes the electronic coherence smaller.

4 Probe-induced active orientation of the sample and forward/backward fragment asymmetry

4.1 Probe-induced active orientation of the sample

As noted in the Methods section of the main text, the ionization rate $W^{(\pm1)}(\hat{\mathbf{R}}, \epsilon, t)$, involved in the averaged value of the probe-induced molecular orientation in the laboratory frame, is

$$W^{(h)}(\hat{\mathbf{R}}, \epsilon, t) \propto \int d\hat{\mathbf{k}} |d^{(h)}_{\mathbf{k}}(\hat{\mathbf{R}}, t)|^2 \tag{13}$$

where $\epsilon = k^2/2$ and $h = \pm 1$. Using equation (5), one obtains

$$\begin{aligned} W^{(h)}(\hat{\mathbf{R}}, \epsilon, t) &= \frac{1}{4\pi} \sum_{i,i',l,m,\nu,\nu'} \mathcal{A}_i(\hat{\mathbf{R}}) \mathcal{A}_{i'}(\hat{\mathbf{R}}) \sqrt{I_{1-NIR}(\omega_i) I_{1-NIR}(\omega_{i'})} \times \\ & \quad D^{(1)}_{\nu,h}(\hat{\mathbf{R}}) D^{(1)^*}_{\nu',h}(\hat{\mathbf{R}}) a_{iklm\nu} a^*_{i'klm\nu'} e^{-i(E_i - E_{i'})t}. \end{aligned} \tag{14}$$

Rearranging the terms in the sum yields

$$\begin{aligned} W^{(h)}(\hat{\mathbf{R}}, \epsilon, t) &= \frac{1}{4\pi} \sum_{i,l,m,\nu,\nu' \geq \nu} \mathcal{A}^2_i(\hat{\mathbf{R}}) I_{1-NIR}(\omega_i) \mathcal{R}[D^{(1)}_{\nu,h}(\hat{\mathbf{R}}) D^{(1)^*}_{\nu',h}(\hat{\mathbf{R}}) a_{iklm\nu} a^*_{iklm\nu'}](2 - \delta_{\nu,\nu'}) \\ &+ \frac{1}{4\pi} \sum_{i,i'>i,l,m,\nu,\nu' \geq \nu} \mathcal{A}_i(\hat{\mathbf{R}}) \mathcal{A}_{i'}(\hat{\mathbf{R}}) \sqrt{I_{1-NIR}(\omega_i) I_{1-NIR}(\omega_{i'})} \mathcal{R}[D^{(1)}_{\nu,h}(\hat{\mathbf{R}}) \times \\ & \quad D^{(1)^*}_{\nu',h}(\hat{\mathbf{R}}) a_{iklm\nu} a^*_{i'klm\nu'} + D^{(1)}_{\nu',h}(\hat{\mathbf{R}}) D^{(1)^*}_{\nu,h}(\hat{\mathbf{R}}) a_{iklm\nu'} a^*_{i'klm\nu}](2 - \delta_{\nu,\nu'}) \cos[(E_i - E_{i'})t] \end{aligned} \tag{15}$$

where the first term corresponds to the time-independent incoherent ($i = i'$) contribution while the second one is associated to the time-dependent coherent ($i \neq i'$) contribution. There is a close link between Equation (15) and Equation (2) of the main text.

We now turn our attention to Equations (5) and (6) of the main text. We choose the molecular frame such that the unitatry $\hat{\mathrm{e}}_{mol}$ vector, along the internal C-C bond of ML, is $\hat{\mathrm{e}}_{mol} = \hat{\mathbf{z}}$.

Therefore, the x-, y- and z- components of $\hat{\mathbf{e}}_{lab}$ are

$$
\begin{aligned}
\hat{\mathbf{e}}_{lab,x}(\hat{\mathbf{R}}) &= (D^{(1)^*}_{0,-1}(\hat{\mathbf{R}}) - D^{(1)^*}_{0,+1}(\hat{\mathbf{R}}))/\sqrt{2} \\
\hat{\mathbf{e}}_{lab,y}(\hat{\mathbf{R}}) &= i(D^{(1)^*}_{0,-1}(\hat{\mathbf{R}}) + D^{(1)^*}_{0,+1}(\hat{\mathbf{R}}))/\sqrt{2} \\
\hat{\mathbf{e}}_{lab,z}(\hat{\mathbf{R}}) &= D^{(1)^*}_{0,0}(\hat{\mathbf{R}}) \qquad (16)
\end{aligned}
$$

Their averaged values over the molecular orientations are, as in Equation (5) of the main text

$$
< \hat{\mathbf{e}}^{(h)}_{lab,\eta} >_{\hat{\mathbf{R}}} (\epsilon, t) = \frac{\int d\hat{\mathbf{R}} W^{(h)}(\hat{\mathbf{R}}, \epsilon, t)\hat{\mathbf{e}}_{lab,\eta}(\hat{\mathbf{R}})}{b_{0_{incoh}}(\epsilon, t)} \qquad (17)
$$

where $\eta = x$, y or z and $b_{0_{incoh}}(\epsilon, t)$ is the incoherent ionization rate, which is associated to the $i = i'$ term in Equation (15) and corresponds to the full ionization rate averaged over time delays t. Using Equation (14), this yields

$$
\begin{aligned}
< \hat{\mathbf{e}}^{(h)}_{lab,\eta} >_{\hat{\mathbf{R}}} (\epsilon, t) &= \frac{1}{4\pi b_{0_{incoh}}(\epsilon, t)} \sum_{i,i',l,m,\nu,\nu'} \sqrt{I_{1-NIR}(\omega_i) I_{1-NIR}(\omega_{i'})} a_{iklm\nu} a^*_{i'klm\nu'} \times \\
& e^{-i(E_i - E_{i'})t} \int d\hat{\mathbf{R}} \mathcal{A}_i(\hat{\mathbf{R}}) \mathcal{A}_{i'}(\hat{\mathbf{R}}) D^{(1)}_{\nu,h}(\hat{\mathbf{R}}) D^{(1)^*}_{\nu',h}(\hat{\mathbf{R}}) \hat{\mathbf{e}}_{lab,\eta}(\hat{\mathbf{R}}). \qquad (18)
\end{aligned}
$$

Rearranging the terms in the sum leads to

$$
\begin{aligned}
< \hat{\mathbf{e}}^{(h)}_{lab,\eta} >_{\hat{\mathbf{R}}} (\epsilon, t) &= \frac{1}{2\pi b_{0_{incoh}}(\epsilon, t)} \sum_{i,i'>i,l,m,\nu,\nu'} \sqrt{I_{1-NIR}(\omega_i) I_{1-NIR}(\omega_{i'})} \mathcal{I}[a_{iklm\nu} a^*_{i'klm\nu'} \times \\
& \int d\hat{\mathbf{R}} \mathcal{A}_i(\hat{\mathbf{R}}) \mathcal{A}_{i'}(\hat{\mathbf{R}}) D^{(1)}_{\nu,h}(\hat{\mathbf{R}}) D^{(1)^*}_{\nu',h}(\hat{\mathbf{R}}) \hat{\mathbf{e}}_{lab,\eta}(\hat{\mathbf{R}})] \sin[E_i - E_{i'})t]. \qquad (19)
\end{aligned}
$$

where no incoherent term (with $i = i'$) survives. $< \hat{\mathbf{e}}^{(h)}_{lab,\eta} >_{\hat{\mathbf{R}}} (\epsilon, t)$ behaves in phase with the electron current which flows within the Rydberg state superposition (Equation (4) of the main text) and drives the orientation process activated by the circularly polarized probe according to propensity.

We explicitly show in Figure S9, using the (3d,4p) two-state expansion detailed in the main text, that: (i) $< \hat{\mathbf{e}}^{(h)}_{lab,x,y} >_{\hat{\mathbf{R}}} (\epsilon, t) = 0$ – this feature holds for all energies ϵ at all delays t; (ii)

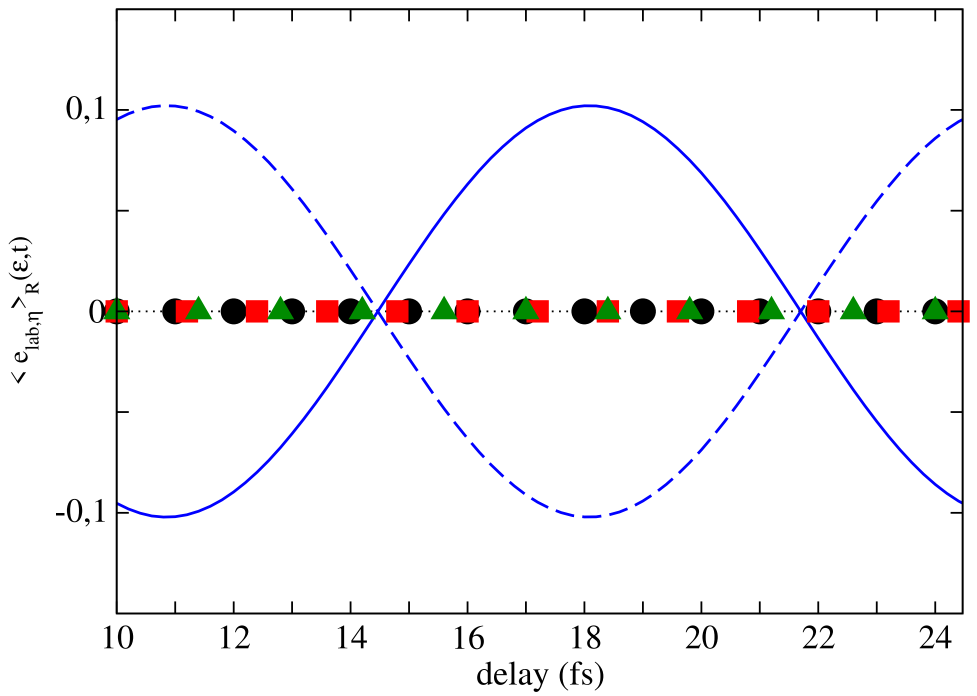

Fig. S9. Averaged orientation of ML cations upon ionization of a 3d-4p Rydberg wavepacket, $< \hat{\mathbf{e}}^{(h)}_{lab,\eta} >_{\hat{\mathbf{R}}} (\epsilon, t)$, for $\epsilon = 0.25$ eV (see eq. (18) and text) and $(h = +1, \eta = z)$: blue line, $(h = -1, \eta = z)$: dashed blue line, $(h = 0, \eta = z)$: black circles, $(h, \eta = x$ or $y)$: red squares, (h, η) for an achiral (H_2) system: green triangles.

$< \hat{\mathbf{e}}^{(-1)}_{lab,z} >_{\hat{\mathbf{R}}} (\epsilon, t) = - < \hat{\mathbf{e}}^{(+1)}_{lab,z} >_{\hat{\mathbf{R}}} (\epsilon, t)$: reversing the probe helicity reverses the asymmetry of the distribution of orientations along the light propagation axis $\hat{\mathbf{z}}$; (iii) $< \hat{\mathbf{e}}^{(0)}_{lab,z} >_{\hat{\mathbf{R}}} (\epsilon, t) = 0$: the chiral features of the electron current induced by the linearly polarized field cannot be imprinted in the distribution of orientations using a linearly polarized probe (as for PECD); (iv) $< \hat{\mathbf{e}}^{(h)}_{lab,z} >_{\hat{\mathbf{R}}} (\epsilon, t) = 0$ for an achiral system – we took H_2 as a prototypical system. All calculations were performed for $\epsilon = 0.25$ eV; the dependence of $< \hat{\mathbf{e}}^{(h)}_{lab,z} >_{\hat{\mathbf{R}}} (\epsilon, t)$ on ϵ will be considered in the next section.

4.2 Directed fragmentation and FBFA

We did not perform an exhaustive study of ML fragmentation. However, we have noted in Section 3 that for trajectories correlating to Isomer2, the central C-C bond of the ML cation

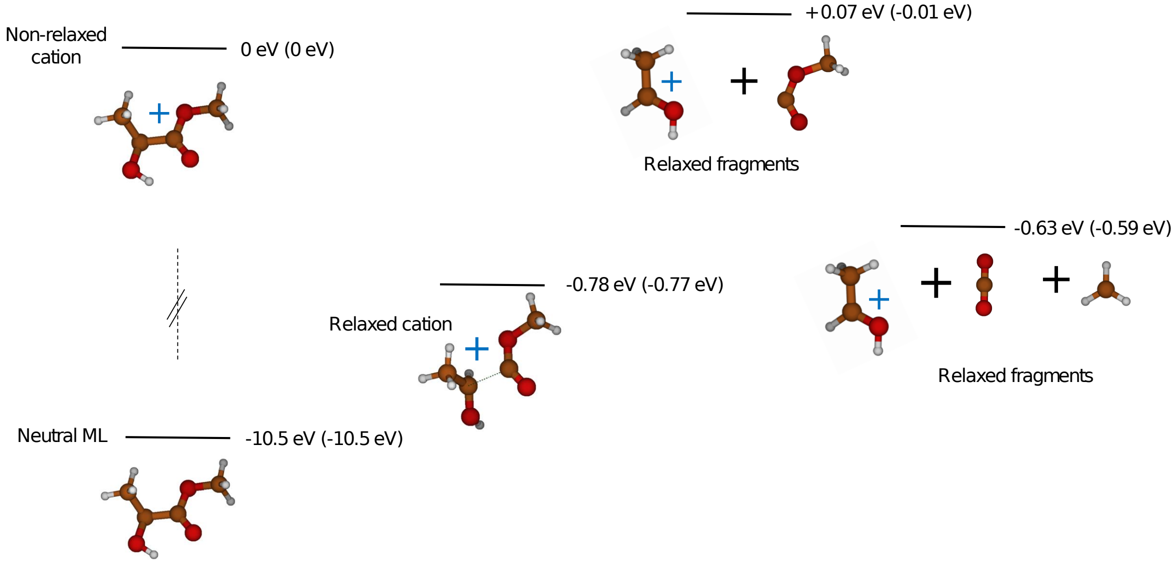

Fig. S10. Energy diagram for the ML cation and associated fragments from its corresponding Isomer2. The energies are obtained by CAM-B3LYP/6-311++G(dp) calculations and compared to energies (in brackets) resulting from CCSD(T) calculations.

elongates during the first tens of femtoseconds subsequently to ionization. Such trajectories are prone to fragmentation $C_4H_8O_3^+ \rightarrow CO_2CH_3 + CH_3CHOH^+$ provided enough energy is placed on vibrational modes involving C-C stretching. The energy requirements are illustrated in Figure S10. The energy of the cation, in the geometry of neutral ML (remaining frozen during the sudden ionization process), is taken as the energy reference. The relaxed cation, *i*.e. Isomer2 at its equilibrium geometry, lies 0.78 eV below the reference. The sum of separate, and relaxed, CO_2CH_3 and CH_3CHOH^+ fragment energies is 0.07 eV larger than the reference. Therefore, two-fragment dissociation is allowed provided 0.07 eV is at least available for C-C stretching. On the other hand, the sum of separate CO_2, CH_3 and CH_3CHOH^+ fragment energies is 0.63 eV below the reference. This indicates that direct three-body fragmentation $C_4H_8O_3^+ \rightarrow CO_2 + CH_3 + CH_3CHOH^+$ is allowed and that the two-step process $C_4H_8O_3^+ \rightarrow CO_2CH_3 + CH_3CHOH^+ \rightarrow CO_2 + CH_3 + CH_3CHOH^+$ is plausible. These energy requirements, derived

from single-point CAM-B3LYP/6-311++G(dp) calculations, have been verified performing single and double (with triple corrections) coupled-cluster (CCSD(T)) calculations (*36*), using an extended atomic natural orbital basis set (*37*). These last calculations, based on higher level of theory than DFT, basically ascertain the former.

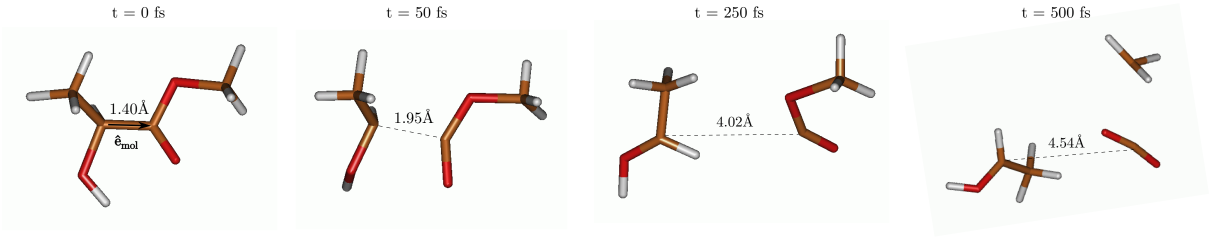

Fig. S11. Illustration of a possible fragmentation process in the ML cation. The time reference ($t = 0$) is the ionization time of neutral ML.

We propagate a few trajectories, that we selected observing an elongating C-C bond at 100 fs, until 1 ps. Most of them seemed to describe the $C_4H_8O_3^+ \rightarrow CO_2CH_3$ + CH_3CHOH^+ process but the fragments stayed quite close to each other, with $R_{C-C} \sim 4$Å, for hundreds of fs, without describing a genuine break-up process. We attribute this lack of dissociation to the classical nature of our calculations and its inherent inability to account for quantum tunneling effects across a potential barrier that may emerge along the trajectory of the reaction. However, we explicitly found the occurence of the two-step process, as illustrated in Figure S11. Such an event is enough to introduce the concept of fragmentation directed by ionization according to propensity. For instance, we see in Figure S9 that for $t \sim 18$ fs and $\epsilon = 0.25$ eV, $< \hat{\mathrm{e}}^{(+1)}_{lab,z} >_{\hat{\mathbf{R}}}$ $(\epsilon, t) \sim 0.1$. This means that the ML cations are preferentially oriented upon ionization with the CH_3CHOH group pointing backward with respect to the light propagation axis $\hat{\mathbf{z}}$ (see Figure S11 where $\hat{\mathrm{e}}_{mol}$ is represented). This preferential orientation will stay encoded in the subsequent fragmentation patterns provided fragmentation occurs on a smaller time scale than rotational dynamics. Here, the $J = 0$ and $J = 1$ rotational constants are ~ 100 ps, which is much larger than the fragmentation timescale illustrated in Figure S11. Therefore, the CH_3CHOH^+ ion

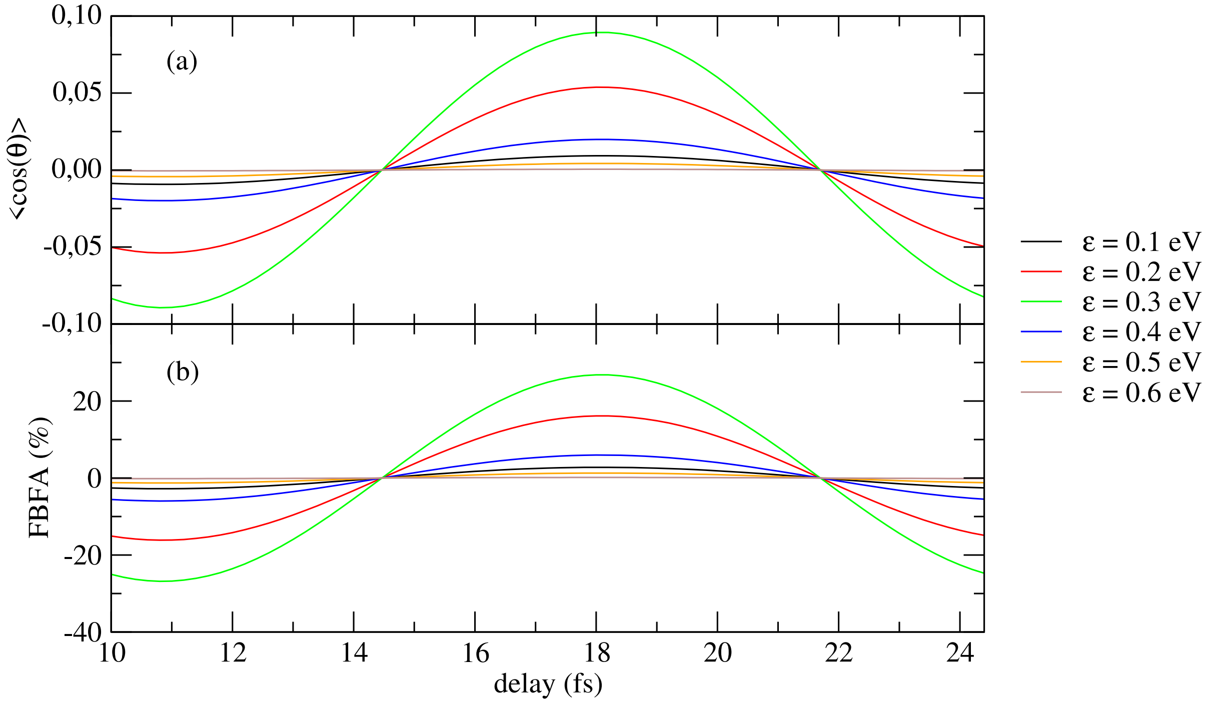

Fig. S12. Dependence on ϵ of (a) $<\cos\theta>^{(+1)}_{\hat{\mathbf{R}}}(\epsilon,t) =< \hat{\mathbf{e}}_{lab,z} >^{(+1)}_{\hat{\mathbf{R}}}(\epsilon,t)$, measuring the preferential orientation along the light propagation axis of ML cations upon ionization and (b) $FBFA^{(+1)}(\epsilon,t)$, the forward/backward fragment asymmetry.

will be preferentially emitted backward while CO_2 and CH_3 rather exit the interaction region through the forward hemisphere. This fragment dichroism can be evaluated quantitatively using the forward/backward fragment asymmetry (FBFA) introduced in the main text. The asymmetry depends on the photoelectron energy, because of ϵ-dependent underlying ionization yields. $\epsilon = 0.25$ eV was considered in the main text. We present in Figure S12 the dependence of both $<\cos\theta>^{(+1)}_{\hat{\mathbf{R}}}(\epsilon,t) =< \hat{\mathbf{e}}_{lab,z} >^{(+1)}_{\hat{\mathbf{R}}}(\epsilon,t)$ and $FBFA^{(+1)}(\epsilon,t)$ on ϵ. Keeping the pump excitation unchanged (leading to the population of the 3d and 4p Rydberg states), ϵ could be varied by changing the wavelength of the probe pulse. $FBFA^{(+1)}(\epsilon,t)$ increases when ϵ increases from 0 to ~ 0.25 eV and decreases quite rapidly for higher photoelectron energies – $FBFA^{(+1)}(\epsilon,t) \sim 0$ for $\epsilon \geq 0.6$ eV. The chiral features of the transient Rydberg wavepacket are lost during high energy ionization processes. However, changing the excitation scheme could lead to higher

FBFA in the same photoelectron energy range.

References

1. Hohenberg, P. & Kohn, W. Inhomogeneous electron gas. *Phys. Rev.* **136**, B864–B871 (1964). URL `https://link.aps.org/doi/10.1103/PhysRev.136.B864`.

2. Dunning, T. H. Gaussian basis functions for use in molecular calculations. i. contraction of (9s5p) atomic basis sets for the first-row atoms. *The Journal of Chemical Physics* **53**, 2823–2833 (1970). URL `https://doi.org/10.1063/1.1674408`.

3. Dunning, T. H. & Hay, P. J. *Gaussian Basis Sets for Molecular Calculations*, 1–27 (Springer US, Boston, MA, 1977). URL `https://doi.org/10.1007/978-1-4757-0887-5`.

4. Becke, A. D. Density-functional thermochemistry. iii. the role of exact exchange. *The Journal of Chemical Physics* **98**, 5648–5652 (1993). URL `https://doi.org/10.1063/1.464913`.

5. Stephens, P. J., Devlin, F. J., Chabalowski, C. F. & Frisch, M. J. Ab initio calculation of vibrational absorption and circular dichroism spectra using density functional force fields. *The Journal of Physical Chemistry* **98**, 11623–11627 (1994). URL `https://doi.org/10.1021/j100096a001`.

6. Schmidt, M. *et al.* General atomic and molecular electronic structure system. *J. Comp. Chem.* **14**, 1347–1363 (1993).

7. Runge, E. & Gross, E. K. U. Density-functional theory for time-dependent systems. *Phys. Rev. Lett.* **52**, 997–1000 (1984). URL `https://link.aps.org/doi/10.1103/PhysRevLett.52.997`.

8. Pritchard, B. P., Altarawy, D., Didier, B., Gibson, T. D. & Windus, T. L. New basis set exchange: An open, up-to-date resource for the molecular sciences community. *Journal of Chemical Information and Modeling* **59**, 4814–4820 (2019). URL https://doi.org/10.1021/acs.jcim.9b00725. PMID: 31600445.

9. Feller, D. The role of databases in support of computational chemistry calculations. *Journal of Computational Chemistry* **17**, 1571–1586 (1996).

10. Schuchardt, K. L. *et al.* *Journal of Chemical Information and Modeling* **47**, 1045–1052 (2007). URL https://doi.org/10.1021/ci600510j. PMID: 17428029.

11. Tawada, Y., Tsuneda, T., Yanagisawa, S., Yanai, T. & Hirao, K. A long-range-corrected time-dependent density functional theory. *The Journal of Chemical Physics* **120**, 8425–8433 (2004). URL https://doi.org/10.1063/1.1688752.

12. Kramida, A., Yu. Ralchenko, Reader, J. & and NIST ASD Team. NIST Atomic Spectra Database (ver. 5.9), [Online]. Available: https://physics.nist.gov/asd [2017, April 9]. National Institute of Standards and Technology, Gaithersburg, MD. (2021).

13. Jungen, M. *Ab Initio Calculations for Rydberg States* (John Wiley and Sons, Ltd, 2011).

14. Zahariev, F. & Gordon, M. S. Nonlinear response time-dependent density functional theory combined with the effective fragment potential method. *The Journal of Chemical Physics* **140**, 18A523 (2014). URL https://doi.org/10.1063/1.4867271.

15. Rose, M. E. *Elementary theory of angular momentum* (John Wiley, 1967).

16. Dill, D. & Dehmer, J. L. Electron-molecule scattering and molecular photoionization using the multiple-scattering method. *The Journal of Chemical Physics* **61**, 692–699 (1974). URL https://doi.org/10.1063/1.1681947.

17. Slater, J. C. & Johnson, K. H. Self-consistent-field $x\alpha$ cluster method for polyatomic molecules and solids. *Phys. Rev. B* **5**, 844–853 (1972). URL https://link.aps.org/doi/10.1103/PhysRevB.5.844.

18. Slater, J. C. A simplification of the hartree-fock method. *Phys. Rev.* **81**, 385–390 (1951). URL https://link.aps.org/doi/10.1103/PhysRev.81.385.

19. Latter, R. Atomic energy levels for the thomas-fermi and thomas-fermi-dirac potential. *Phys. Rev.* **99**, 510–519 (1955). URL https://link.aps.org/doi/10.1103/PhysRev.99.510.

20. Johnson, B. R. The renormalized numerov method applied to calculating bound states of the coupled-channel schroedinger equation. *The Journal of Chemical Physics* **69**, 4678–4688 (1978). URL https://doi.org/10.1063/1.436421.

21. Goetz, R. E., Isaev, T. A., Nikoobakht, B., Berger, R. & Koch, C. P. Theoretical description of circular dichroism in photoelectron angular distributions of randomly oriented chiral molecules after multi-photon photoionization. *The Journal of Chemical Physics* **146**, 024306 (2017). URL https://doi.org/10.1063/1.4973456.

22. Bransden, B. H. & Joachain, C. J. *Physics of Atoms and Molecules; 2nd ed.* (Prentice-Hall, Harlow, 2003). URL https://cds.cern.ch/record/1095023.

23. Lehmann, C. S., Ram, N. B., Powis, I. & Janssen, M. H. M. Imaging photoelectron circular dichroism of chiral molecules by femtosecond multiphoton coincidence detection. *The Journal of Chemical Physics* **139**, 234307 (2013). URL https://doi.org/10.1063/1.4844295.

24. Blanchet, V. *et al.* Ultrafast relaxation investigated by photoelectron circular dichroism: an isomeric comparison of camphor and fenchone. *Phys. Chem. Chem. Phys.* **23**, 25612–25628 (2021). URL `http://dx.doi.org/10.1039/D1CP03569J`.

25. Powis, I. *Photoelectron Circular Dichroism in Chiral Molecules*, chap. 5, 267–329 (John Wiley and Sons, Ltd, 2008).

26. Yanai, T., Tew, D. P. & Handy, N. C. A new hybrid exchange–correlation functional using the coulomb-attenuating method (cam-b3lyp). *Chemical Physics Letters* **393**, 51–57 (2004).

27. Krishnan, R., Binkley, J. S., Seeger, R. & Pople, J. A. Self-consistent molecular orbital methods. xx. a basis set for correlated wave functions. *The Journal of Chemical Physics* **72**, 650–654 (1980). URL `https://doi.org/10.1063/1.438955`.

28. Clark, T., Chandrasekhar, J., Spitznagel, G. W. & Schleyer, P. V. R. Efficient diffuse function-augmented basis sets for anion calculations. iii. the 3-21+g basis set for first-row elements, li–f. *Journal of Computational Chemistry* **4**, 294–301 (1983). URL `https://onlinelibrary.wiley.com/doi/abs/10.1002/jcc.540040303`.

29. Barbatti, M. *et al.* Newton-x: a surface-hopping program for nonadiabatic molecular dynamics. *WIREs Computational Molecular Science* **4**, 26–33 (2014).

30. Barbatti, M. *et al.* Newton-x: A package for newtonian dynamics close to the crossing seam (v. 2.2). available via the internet at www.newtonx.org (2018).

31. Frisch, M. J. *et al.* Gaussian09 revision e.01. Gaussian Inc. Wallingford CT 2009.

32. Golubev, N. V., Begušić, T. & Vaníček, J. c. v. On-the-fly ab initio semiclassical evaluation of electronic coherences in polyatomic molecules reveals a sim-

ple mechanism of decoherence. *Phys. Rev. Lett.* **125**, 083001 (2020). URL https://link.aps.org/doi/10.1103/PhysRevLett.125.083001.

33. Arnold, C., Vendrell, O. & Santra, R. Electronic decoherence following photoionization: Full quantum-dynamical treatment of the influence of nuclear motion. *Phys. Rev. A* **95**, 033425 (2017). URL https://link.aps.org/doi/10.1103/PhysRevA.95.033425.

34. Matselyukh, D., Despré, V., Golubev, N., Kuleff, A. & Wörner, H. J. Decoherence and revival in attosecond charge migration driven by non-adiabatic dynamics (2021). URL https://arxiv.org/abs/2110.00357.

35. Keefer, D., Schnappinger, T., de Vivie-Riedle, R. & Mukamel, S. Visualizing conical intersection passages via vibronic coherence maps generated by stimulated ultrafast x-ray raman signals. *Proceedings of the National Academy of Sciences* **117**, 24069–24075 (2020). URL https://www.pnas.org/doi/abs/10.1073/pnas.2015988117.

36. Watts, J. D., Gauss, J. & Bartlett, R. J. Coupled-cluster methods with noniterative triple excitations for restricted open-shell Hartree–Fock and other general single determinant reference functions. Energies and analytical gradients. *The Journal of Chemical Physics* **98**, 8718–8733 (1993). URL https://doi.org/10.1063/1.464480.

37. Widmark, P., Malmqvist, P. & Roos, B. Density matrix averaged atomic natural orbital (ano) basis sets for correlated molecular wave functions. *Theoret. Chim. Acta* **77**, 291–306 (1990).
